# Spatial clustering of measles vaccination coverage among children in sub-Saharan Africa

**DOI:** 10.1186/s12889-017-4961-9

**Published:** 2017-12-15

**Authors:** Tenley K. Brownwright, Zan M. Dodson, Willem G. van Panhuis

**Affiliations:** 1 Department of Epidemiology, University of Pittsburgh Graduate School of Public Health, 130 DeSoto Street, 715 Parran Hall, Pittsburgh, PA 15261 USA; 2 Department of Health Policy and Management, University of Pittsburgh Graduate School of Public Health, 130 DeSoto Street, 702 Parran Hall, Pittsburgh, PA 15261 USA

**Keywords:** Africa, MCV, Measles, Spatial analysis, Spatial heterogeneity, Spatial regression

## Abstract

**Background:**

During the past two decades, vaccination programs have greatly reduced global morbidity and mortality due to measles, but recently this progress has stalled. Even in countries that report high vaccination coverage rates, transmission has continued, particularly in spatially clustered subpopulations with low vaccination coverage.

**Methods:**

We examined the spatial heterogeneity of measles vaccination coverage among children aged 12–23 months in ten Sub-Saharan African countries. We used the Anselin Local Moran’s I to estimate clustering of vaccination coverage based on data from Demographic and Health Surveys conducted between 2008 and 2013. We also examined the role of sociodemographic factors to explain clustering of low vaccination.

**Results:**

We detected 477 spatial clusters with low vaccination coverage, many of which were located in countries with relatively high nationwide vaccination coverage rates such as Zambia and Malawi. We also found clusters in border areas with transient populations. Clustering of low vaccination coverage was related to low health education and limited access to healthcare.

**Conclusions:**

Systematically monitoring clustered populations with low vaccination coverage can inform supplemental immunization activities and strengthen elimination programs. Metrics of spatial heterogeneity should be used routinely to determine the success of immunization programs and the risk of disease persistence.

## Background

Measles is a highly contagious viral disease and is one of the leading causes of death among children in low-income countries, accounting for 114,900 deaths globally in 2014 of which 73,914 (63%) occurred in Africa [1, 2]. Measles also continues to cause epidemics in high-income countries, despite the availability of a safe and highly efficacious vaccine [3, 4].

The Measles-Rubella Initiative, spearheaded by the American Red Cross, the US Centers for Disease Control and Prevention (CDC), the World Health Organization (WHO), and others, has targeted the measles virus for global elimination. This initiative aims to reduce annual measles incidence rates (IRs) to less than five cases per million, requiring >90% coverage of at least one dose of measles containing vaccine (MCV), recommended at age 12 months, by the end of 2015 and >95% coverage by 2020 in all countries [5]. Improvement in vaccination coverage has decreased measles deaths from over half a million globally in 2000 to 114,900 in 2014 [1]. Since 2010, however, progress has stalled [1]: The 2015 vaccination goal was not met and IRs remained relatively unchanged between 2013 and 2014 [1].

Measles elimination is complicated by the high transmission rate of the measles virus. This transmission rate can be expressed as the basic reproductive rate (R_0_), defined as the number of infections caused, on average, by one infectious person in a fully susceptible population [6]. The R_0_ for measles ranges from 15 to 20 infections, which is one of the highest among all infectious diseases (e.g., influenza has an R_0_ around 1.5–2.0) [7]. This high R_0_ leads to the very high critical vaccination fraction for measles of 95%, i.e., the vaccination coverage needed for herd immunity [8]. This critical vaccination fraction assumes that vaccination coverage and population mixing are distributed homogeneously throughout a country [9]. This assumption of homogeneity is not always realistic, as recently found in Mozambique [10] and Malawi [11]. Spatial heterogeneity of vaccination coverage can increase the critical vaccination fraction required for herd immunity to a level exceeding the 95% coverage goal set by the Measles-Rubella Initiative [12, 13].

Spatial heterogeneity of vaccination coverage can delay disease elimination, as illustrated by continued measles outbreaks, even in countries with high average nationwide vaccination coverage rates [14, 15]. Substantial heterogeneity in measles vaccination coverage has been demonstrated previously in Sub-Saharan Africa [16], but drivers for this heterogeneity are poorly understood. Timely detection and targeting of low-coverage population clusters by supplemental immunization activities (SIAs) can lead to protective herd immunity and accelerate disease elimination, as demonstrated by the successful strategy used for measles elimination in the Americas [17]. We used publicly available microdata from the Demographic and Health Surveys (DHS) in Sub-Saharan Africa to determine if subpopulations with low vaccination coverage are clustered and explore possible determinants.

## Methods

### Clustering algorithm

We collected measles vaccination coverage data from the most recent DHS conducted in ten countries: Burundi in 2010; the Democratic Republic of the Congo (DRC) in 2013; Kenya in 2008; Madagascar in 2008; Malawi in 2010; Mozambique in 2011; Rwanda in 2010; Tanzania in 2010; Zambia in 2013–14; and Zimbabwe in 2010–11 [18]. These countries were selected based on their contiguity and data availability. We obtained approval from DHS to download and use these data for this study.

DHS are nationwide surveys that are representative of the population and detailed survey methodology has been published elsewhere [19]. In short, DHS are performed using a two-stage cluster sampling design: In the first stage, the DHS selects a random sample of clusters (groups of possible sample households in close proximity to each other) from an already existing sample frame (e.g., a population census); in the second stage, a random sample of households is selected within each cluster. The DHS also determines sample weights that should be applied to survey data to ensure that all subpopulations are equally represented [20]. We extracted, from DHS, the vaccination status (first dose MCV) of children aged 12–23 months measured from sampled households in each cluster, not differentiating between vaccine doses received from routine immunization or SIAs. In the DHS, vaccination status is obtained from vaccination cards where possible and otherwise from mothers’ reports [20]. We calculated the cluster-level vaccination coverage rate as the median of the weighted household-level vaccination coverage rates.

We estimated the spatial association of MCV coverage rates among DHS clusters using the Global Moran’s I and Anselin Local Moran’s I statistics. The Global Moran’s I ranges from −1 to 1 and is a single estimate of spatial association among all DHS clusters (spatial autocorrelation). Values close to zero indicate the absence of a spatial association (i.e., a random distribution), values close to negative one indicate strong spatial dispersion, and values close to positive one indicate strong clustering (autocorrelation). The Anselin Local Moran’s I estimates the association of vaccination coverage rates between a DHS cluster and its neighboring clusters within a specified geographical area (inter-cluster variation). The Anselin Local Moran’s I has been used previously for similar analyses to locate pockets of childhood stunting in Nigeria [21]. We used the Anselin Local Moran’s I to estimate spatial clustering of low (< 75%), high (≥ 75%), or mixed (low near high or vice versa) weighted vaccination coverage. We considered Moran’s I statistics with *p*-values < 0.05 to be statistically significant. DHS cluster data have been used previously for similar clustering analyses, such as examining malnutrition in Ethiopia [22], of HIV prevalence in Burundi [23], and of childhood stunting in Nigeria [24].

### Determinants of low-vaccination clusters

We explored possible determinants for clustering of low-vaccination using additional information from country DHS: (1) child in possession of a health card or not (*H*_*c*_); (2) mother had heard of oral rehydration salts (ORS) or not (*O*); (3) mother is literate or not (*T*); (4) mother visited a health facility in the last 12 months or not (*H*_*f*_); (5) mother mentioned that money had been a barrier to seeking healthcare in the past or not (*M*). We calculated the cluster-level percent children with a health card as the median of the weighted household-level percentages of children with a health card. From the other household-level variables, we computed the cluster-level equivalents as the percent of mothers (households) that answered each question affirmatively.

We used a logistic regression model to estimate the association between the odds for a DHS cluster being part of a low-vaccination spatial cluster and the aforementioned explanatory factors. We adjusted for spatial autocorrelation (inter-cluster variation) of vaccination status among clusters with a queen contiguity weights matrix based on spatial lags. Queen contiguity calculates spatial autocorrelation of the outcome variable among all contiguous neighbors, after creating Thiessen polygons around cluster coordinates [25–27]. We selected a contiguity weights matrix instead of a distance matrix due to the large variability in distances between clusters in our ten-country study area.

Our model took the following form:

$$ \ln \left(\frac{L(x)}{1-L(x)}\right)=\rho W\ln \left(\frac{L(x)}{1-L(x)}\right)+{\beta}_0+{\beta}_1{H}_c+{\beta}_2O+{\beta}_3T+{\beta}_4{H}_f+{\beta}_5M+\varepsilon $$

where $\left(\frac{L(x)}{1-L(x)}\right)$ represented the odds of being in a low-vaccination spatial cluster, *ρ* represented the spatial autoregressive coefficient for the log odds of being in a low-vaccination spatial cluster, *W* represented the queen contiguity matrix, *β*_*1–5*_ represented the regression coefficients for aforementioned covariates, and *ε* represented the error term.

We used SAS version 9.4 and ArcGIS version 10.4 for this analysis.

## Results

### Country-level vaccination coverage

**Fig. 1:**
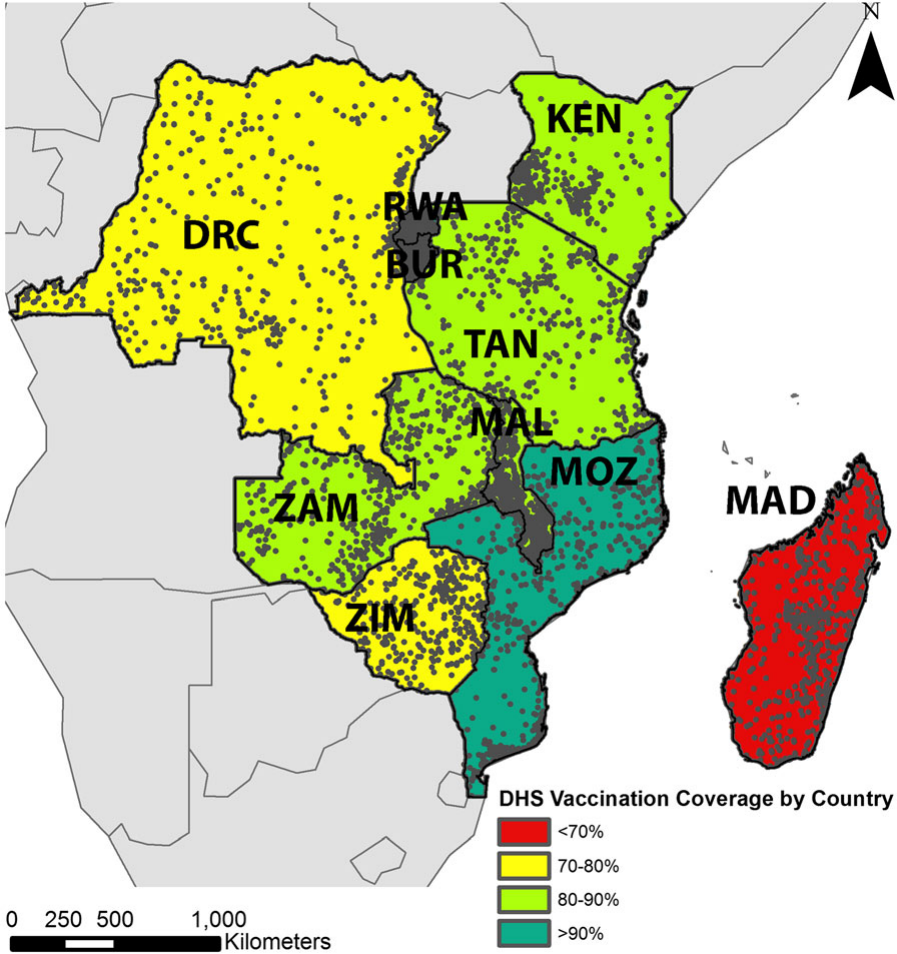
Vaccination coverage and DHS clusters in the study area. The location of each DHS cluster is depicted as a grey circle. We computed the average vaccination coverage rate for each country from DHS cluster-level data. Both maps were created by study investigators using open access data sources

We included a total of 5458 DHS clusters containing 70,092 households across all ten countries (Fig. 1, Table 1). This sample is representative of a total population of 214,339,000 people. Nationwide MCV coverage among children aged 12–23 months was below the measles critical vaccination fraction of 95% for nine out of the ten countries and ranged from a low of 69.6% for Madagascar to a high of 95% for Rwanda. The average MCV coverage for all 10 countries, weighted by population size, was 83.6%.

**Table 1:** DHS survey populations included by country

Country	Survey year	Clusters	House-holds	Population^a^ in sampled households	Population in all households (1000’s)	MCV^b^ coverage (%)
Burundi	2010	376	4662	7742	9233	94.3
DRC	2013–2014	536	10,023	18,716	67,514	71.6
Kenya	2008–2009	398	3864	6079	3877	85.0
Madagascar	2008–2009	595	8151	12,448	19,927	69.6
Malawi	2010	849	12,889	19,967	15,014	93.0
Mozambique	2011	610	6882	11,102	24,581	81.5
Rwanda	2010	492	6019	9002	10,837	95.0
Tanzania	2010	475	4862	8023	44,973	84.5
Zambia	2013–2014	721	8692	13,457	14,539	84.9
Zimbabwe	2010–2011	406	4048	5564	13,077	79.1
Total		5458	70,092	112,100	214,339	83.6

Legend: ^a^children 12–23 months of age, ^b^Measles containing vaccine

### Clustering of low vaccination coverage rates

**Fig. 2:**
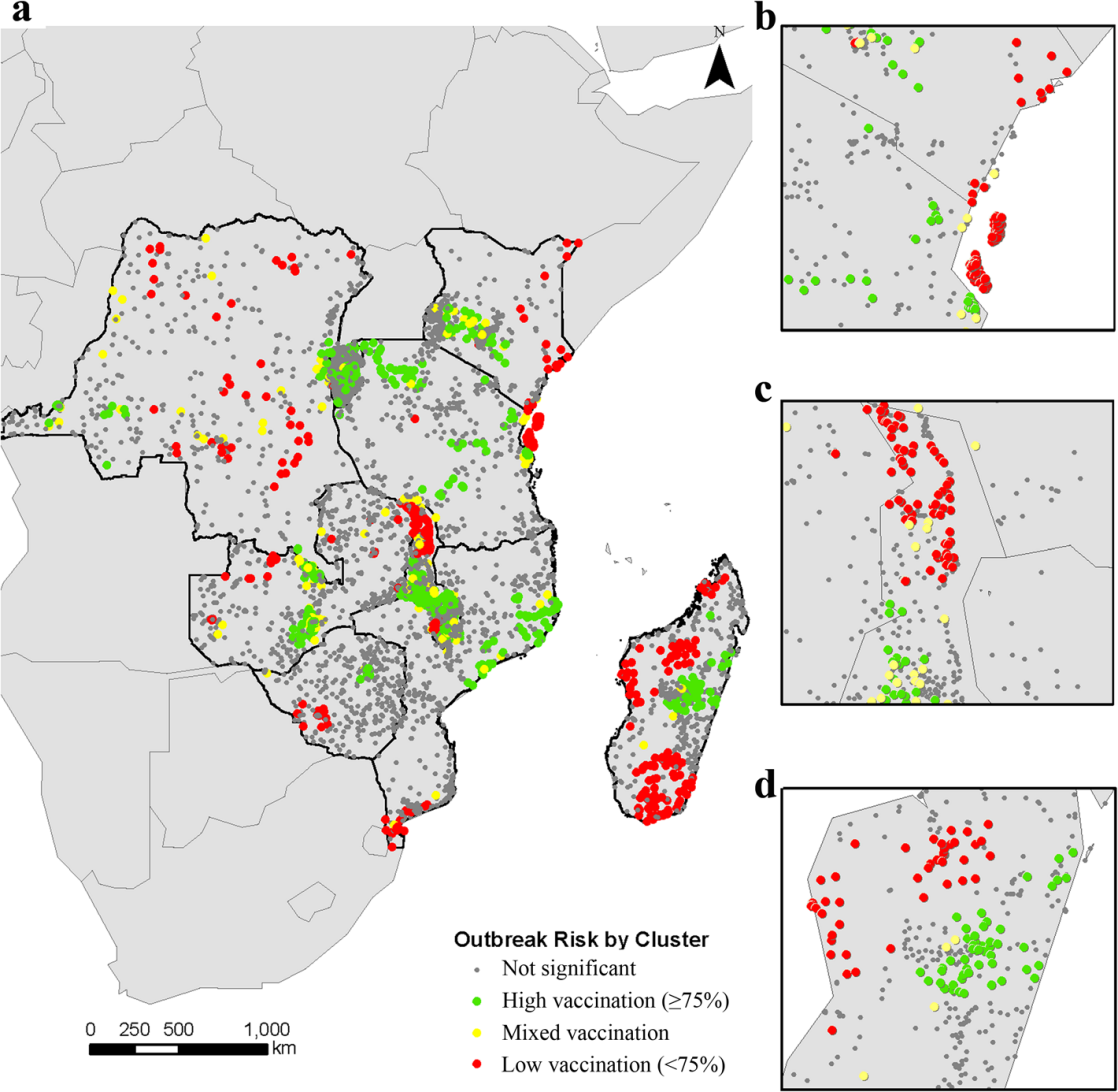
Spatial clustering of vaccination coverage in DHS clusters. Using the Anselin Local Moran’s I, we classified each DHS cluster as being part of a spatial cluster with low-vaccination, high-vaccination, or mixed vaccination coverage (low-vaccination near high-vaccination or vice versa). Grey circles indicate that vaccination coverage for a DHS cluster was not statistically significantly clustered. **a** We detected clustering of low, mixed, and high vaccination coverage in all countries. Vaccination coverage in some spatial clusters contrasted nationwide vaccination coverage rates: e.g., **b** in the Zanzibar/Pemba islands and the Kenya-Tanzania border population (low vaccination vs. high nationwide); **c** in Northern Malawi (low vaccination vs. high nationwide); and (**d**) in the Madagascar capital region (high vaccination vs. low nationwide)

We found strong spatial heterogeneity in measles vaccination coverage across the entire ten-country region (Global Moran’s I of 0.388, *p* < 0.001). Based on the Anselin Local Moran’s I, we identified statistically significant spatial correlation of low vaccination coverage (< 75%) between 477 DHS clusters, of mixed coverage between 148 clusters, and of high coverage (≥ 75%) between 645 clusters (Fig. 2a). The DRC had the second-lowest nationwide vaccination coverage rate in our study region and had clustering of low coverage throughout the country. We found clustering of high coverage almost uniformly throughout Rwanda and Burundi, two countries with the highest nationwide average vaccination coverage in our sample. In other countries, clustering of low-coverage was concentrated in specific geographic areas: e.g., East Kenya, North Malawi, North Zambia, South Zimbabwe, and South Mozambique. Madagascar had the lowest average nationwide MCV coverage in our sample and had clustering of low-coverage throughout the country except in the capital region. We also found clustering of low coverage across the Kenya-Tanzania and the Malawi-Zambia borders.

We found three areas with clustering of vaccination coverage that contrasted nationwide average rates: (1) the Zanzibar/Pemba island population in Kenya-Tanzania had clustering of low coverage while nationwide rates were relatively high (Fig. 2b); (2) the Madagascar capital region had clustering of high coverage while the nationwide rates were low (Fig. 2c); and (3) the Northern Malawi region had clustering of low vaccine coverage compared to high nationwide coverage (Fig. 2d). Each of these areas has distinctive geological features that separated them from the surrounding area: Zanzibar and Pemba are Tanzanian island-populations with semi-autonomous governments; the Madagascar capital region of Antananarivo is located in the mountainous Hauts Plateaux region, separated from the rest of the country; and Northern Malawi includes most of Lake Malawi and areas of higher elevation compared to the South of the country that includes the river Shire.

### Determinants of low-vaccination

We explored possible determinants for clustering of low vaccination coverage using a spatial regression model. Clustering of low vaccination coverage was associated with children not having a health card and mothers not having knowledge of ORS. Clustering of low vaccination was 4.6% less likely for each percentage point increase in children with a health card (95% CI: -0.066, −0.026, *p* < 0.01) and 1.7% less likely for each percentage point increase in mothers with knowledge of ORS (95% CI: -0.033, −0.001, *p* < 0.05). In addition, clustering of low coverage was inversely related to having financial restrictions to healthcare, where we found 1.6% less likely per percentage point increase in mothers listing financial barriers to seeking healthcare (95% CI: -0.029, −0.003, *p* < 0.05). Maternal literacy rates and a maternal history of visiting a health clinic were not statistically significantly associated with clustering of low vaccination coverage.

## Discussion

Using publicly available DHS data from 10 countries, we found 477 geographical clusters of low measles vaccination coverage spread across Sub-Saharan Africa, many of which contrasted relatively high nationwide average vaccination coverage rates. These clusters can weaken herd immunity, cause inequity in disease risk, and delay elimination programs. Indeed, recent measles outbreaks have occurred in subpopulations with low immunization rates: Zambia had an average MCV coverage of 84.9%, and Malawi of 93%, but both countries experienced a large measles outbreak in 2010–2011 [28]; this outbreak spread from high-risk subpopulations in South Africa to Zambia, Malawi, and to high-risk subpopulations in Tanzania consistent with the clusters that we identified [29, 30]. The persistence of virus transmission due to highly connected, clustered, unimmunized subpopulations has also been demonstrated by mathematical metapopulation models [31–33]. These models can be used to compute vaccination coverage goals that take into account spatial clustering of low vaccination.

We found that clustering of low vaccination coverage was more likely in populations with low health education and with limited access to healthcare. Previous studies have found similar risk factors for low immunization rates [11, 34, 35]. We also found that financial barriers to healthcare were associated with better vaccination rates, which seems counterintuitive. One possibility for this observed relationship may be that vaccination is often free of charge and may not be affected by financial barriers. In Malawi, for example, high vaccine uptake was observed despite significant cost and travel time, possibly related to high levels of trust in the effectiveness of the vaccine to prevent serious disease [36]. In high-income countries, indirect measures of wealth have been found to correlate with decreased vaccination coverage due to vaccination hesitancy [37], but income-related vaccination hesitancy has not been found (yet) in Sub-Saharan Africa.

Subpopulations with low vaccination coverage across country borders are a particular concern for measles elimination because these transient populations are often not covered by national immunization programs [10, 16, 17]. We found such subpopulations at the Kenya-Tanzania border and the Malawi-Zambia border. The Kenya-Tanzania border area includes the famous Serengeti and Kilimanjaro national parks and is inhabited by the nomadic Maasai people, who have among the lowest vaccination coverage in Tanzania due to lower use of healthcare services [38, 39]. The Malawi-Zambia border is crossed frequently by the Chewa people that reside in both countries, though this group has been found to be no less vaccination-compliant than other groups of similar socioeconomic status in the region [40, 41]. Trans-border populations with low vaccination coverage can be especially vulnerable to disease importations from one country into another. Such importations occurred during the 2010–2011 measles outbreak that spread from Malawi into Zambia [28]. Coordination of immunization activities between countries will be essential to increase coverage and eliminate measles in these cross-border populations [17].

Most countries in our sample had vaccination coverage rates well below the measles critical vaccination fraction and have already been identified by the Measles-Rubella Initiative as high priority areas for continued activities to increase immunization rates [42]. We found strong spatial heterogeneity of vaccination coverage in some of these countries, indicating that the nationwide vaccination coverage target of 95% set by the Measles-Rubella Initiative may not lead to herd immunity, but that targeted SIAs will be necessary to reach particularly vulnerable populations. For this reason, the Measles-Rubella Initiative also monitors vaccination coverage at the district level.

## Conclusions

Even in countries where national average vaccination coverage rates approach the critical vaccination fraction for measles, subnational vaccination coverage can fall short, leaving subpopulations, including those that cross country borders, vulnerable to outbreaks. Systematically identifying and monitoring these low vaccination sub-populations can inform SIAs and strengthen elimination programs. In addition to monitoring average vaccination coverage statistics, metrics of spatial heterogeneity should be used to determine the success of immunization programs and the risk of disease persistence.

## Abbreviations

CDC: Centers for Disease Control and Prevention
DHS: Demographic and Health Surveys
DRC: Democratic Republic of the Congo
IR: Incidence rate
MCV: Measles containing vaccine
ORS: Oral rehydration salts
SIA: Supplemental immunization activity
WHO: World Health Organization
